# Nonadherence to antiasthmatic medications and its predictors among asthmatic patients in public hospitals of Bahir Dar City, North West Ethiopia: using ASK-12 tool

**DOI:** 10.1186/s40733-023-00091-1

**Published:** 2023-05-04

**Authors:** Teshome Bitew Demelash, Getahun Asmamaw, Liknaw Workie Limenh, Yeniewa Kerie Anagaw, Wudneh Simegn, Wondim Ayenew

**Affiliations:** 1 Department of Pharmaceutics and Social Pharmacy, Addis Ababa University, Addis Ababa, Ethiopia; 2 Department of Pharmacy, Arba Minch University, Arba Minch, Ethiopia; 3 Department of Pharmaceutics, University of Gondar, Gondar, Ethiopia; 4 Department of Pharmaceutical Chemistry, University of Gondar, Gondar, Ethiopia; 5 Department of Social and Administrative Pharmacy, University of Gondar, Gondar, Ethiopia

**Keywords:** Non-adherence, Asthma, Bahir Dar, Ethiopia

## Abstract

**Background:**

Globally, adequate asthma control is not yet achieved. The main cause of uncontrollability is nonadherence to prescribed medications.

**Objectives:**

The objective of this study is to assess asthmatic patients' non-adherence to anti-asthmatic medications and the predictors associated with non-adherence.

**Methods:**

An institution-based cross-sectional study was conducted in three governmental hospitals in Bahir Dar city from September 5 to December 12, 2021. The data was collected using the Adherence Starts with Knowledge-12 tool (ASK-12). Systematic random sampling was applied to select study participants. Bivariable and multivariable logistic regression analyses were used to identify predictors of non-adherence. All statistical tests were analyzed using STATA version 16. *P*-values less than 0.05 were considered statistically significant.

**Results:**

A total of 422 asthmatic patients were included in the study. Most of the study participants (55.4%) did not adhere to their prescribed anti-asthmatic medicines. The educational status of the study participants (AOR = 0.03, 95% CI = 0.00–0.05), family history of asthma (AOR = 0.13, 95% CI = 0.04–0.21), and disease duration that the patients were living with (AOR = 0.01, 95% CI = 0.00–0.01) were the predictors of non-adherence to anti-asthmatic medications.

**Conclusions:**

The level of nonadherence to treatment among patients with asthma was high. Religion, educational status of study participants, family history of asthma, and duration of the disease were the predictors of non-adherence of asthmatic patients to their antiasthmatic medications. Therefore, the Ministry of health, health policy makers, clinicians, and other healthcare providers should pay attention to strengthening the adherence level to antiasthmatic medications, and country-based interventions should be developed to reduce the burden of non-adherence to anti-asthmatic medications.

## Background

Asthma is a chronic, heterogeneous respiratory disease characterized by different symptoms such as shortness of breath, coughing, and wheezing. It is a major health problem that affects more than 339 million people worldwide [1]. There has been an increase in the economic burden, morbidity, and mortality of the disease in the last four decades. Asthma ranked around 28^th^ among disease leading causes of the burden worldwide and 27^th^ in low- and middle-income countries [2]. A recent study suggested that, in European countries, particularly the United Kingdom, asthma-associated deaths increased by 33% between 2008 and 2018 [3]. It is also the most common chronic illness in different African countries; for example, South Africa is ranked 25^th^ in the world for prevalence of asthma and is also ranked fifth for asthma mortality, with approximately 18.5 deaths per 100,000 asthma cases [4]. Even though Ethiopia signed on to achieve the sustainable development goal of reducing deaths from non-communicable diseases by one-third from 2016 to 2030, annual deaths due to non-communicable diseases such as asthma were still high (39%) [5]. The World Health Organization (WHO) report indicated that, in Ethiopia, 1.12% of the total deaths were caused by asthma, and it is also ranked 18^th^ in the world [6].

Even though recent clinical guidelines recommend asthma control as the main therapeutic goal and multiple prevention, diagnostic, and therapeutic strategies have been implemented, adequate asthma control is still unattainable in many countries around the globe [7]. Nonadherence to prescribed medications is the most common cause of asthmatic uncontrollability [8]. Adherence is defined as "the degree to which patients’ behavior corresponds to the recommendations from health care providers" [9]. Non-adherence to prescribed medications in patients with asthma is an important cause of the disease's uncontrollability. The rate of medication non-adherence in patients with asthma ranged from 30 to 70% [10]. The annual economic burden of asthma in different developing countries was more than $20 billion and represented up to three-quarters of the total costs, mainly associated with uncontrolled asthma [11, 12]. Additionally, nonadherence to inhaled corticosteroids is likely responsible for 24% of asthma exacerbations [13]. Low levels of asthma control result in decreased quality of life, increased health care utilization, increased number and length of hospital admissions, increase lost productivity, and an increase in mortality [14].

Several predictors can be related to the patient, the disease, treatment, or physician–patient relationship that have been identified with nonadherence to medication in patients with asthma and other chronic diseases [15]. According to self-regulation theory, perception of illness, treatment beliefs, patient behavior, and forgetfulness are highly associated with medication non-adherence [16]. In addition to these, other factors such as the cost of therapy, the perceived efficacy of medicines, and complex dosing regimens are known to positively or negatively affect treatment adherence in patients with asthma and other chronic diseases [17].

Assessing treatment beliefs, forgetfulness, and inconvenience, including behaviour about asthma medication, may help to identify patients with nonadherence to treatment in clinical practice. These would also guide additional attention, helping to increase the likelihood of appropriate use of asthmatic medications. However, only limited information is available regarding treatment beliefs, forgetfulness, inconvenience, and behavior in Ethiopian asthma patients. Furthermore, evidence identifying forgetfulness, inconvenience, patient behaviour, and treatment belief factors associated with asthma medication nonadherence among the Ethiopian population is not currently available. Therefore, this study aimed to assess the nonadherence of asthmatic patients to anti-asthmatic medications and predictors associated with nonadherence using the ASK-12 tool.

## Methods

### Study design and population

The study was conducted in the outpatient departments and chest clinics of three public hospitals in Bahir Dar city from September 5 to December 12, 2021. These three public hospitals are Tibebe Ghion Specialised Hospital (TGSH), Felege-Hiwot Comprehensive Specialized Hospital (FHCSH), and Addis-Alem Hospital. The hospitals are located in different parts of the city. The study design was an institution-based cross-sectional study design. Asthmatic patients whose age was 18 years and older, who were on active follow-up, who took asthma medication for at least 6 months before enrolment, and patients who can speak and understand the Amharic language were included in the study. Patients with asthma who are critically ill, patients who have acutely exacerbated asthma, and asthmatic patients who did not volunteer to participate in the study or who did not complete the questionnaire were excluded from the study.

### Sample size determination and sampling technique

The sample size of the study was determined using the single population formula.

$$n= \frac{{Z}^{2 }P(1-P)}{{d}^{2}}$$

where n is the sample size required; Z, the degree of accuracy required at a 95% confidence level, is 1.96; P is the prevalence rate of poor glycemic control; and d is the margin of error of 5% (d = 0.05). Reviewing different previous studies, we took the 50% prevalence rate of drug nonadherence, which gives the largest sample size and representativeness of the sample that finally ensures generalization and precision of the sample used in the study.

$$n= \frac{{1.96}^{2 }0.5(1-0.5)}{{0.05}^{2}}=384$$

For the possible non-response rate, 10% of the calculated sample was added to obtain the final sample size of 422 asthmatic patients. A systematic random sampling technique was used for the recruitment of study participants. The total number of asthma patients who had follow-up at the chest clinics of the three government hospitals were 2030. From the total number of asthmatic patients who have active follow-up in the three government hospitals, 30 respondents were excluded because they were selected for the pre-test of the study. Therefore, the remaining 2000 asthmatic patients (470 in TGSH, 1000 at FHCSH, and 530 in Addis-Alem Hospital) were used as a sampling frame to select all study participants. Then, dividing the product of total asthmatic patients in each hospital and sample size by sampling frame, $\left(\frac{470x422}{2000}=99,\frac{530x422}{2000}=112,\;and\;\frac{1000x422}{2000}=211\right)$ were proportionally taken from TGSH, Addis-Alem hospital and FHCSH respectively. The sampling interval was calculated by dividing the number of patients at follow-up by the sample size of the study $\left(\frac{2000}{422}=4\right)$. The first participant was selected by the lottery method from the list prepared based on the number of their medical records. These numbers of medical records were taken from the registration books of each hospital. After that, every fourth participant was recruited for the study.

### Data collection instrument

A data abstraction format was prepared for sociodemographic characteristics, disease, and medical history of patients was prepared by reviewing different literatures. We also used adapted ASK-12 questioners. The ASK-12-Am is a 12-item, self-administered questionnaire consisting of three domains related to medication adherence. These domains are inconvenience or forgetfulness, treatment beliefs, and behaviour. Three items (1, 2, and 3) are related to inconvenience or forgetfulness; four items (4, 5, 6, and 7) are related to treatment belief; and the remaining five items (8, 9, 10, 11, and 12) are related to behavior. Responses with a higher score indicate greater difficulties adherence. Item numbers 4, 5, 6 and 7 were reversibly scored. Then, their final scores were the same as the other 8 items [18]. The total score can range from 12 to 60, and the higher score representing a greater barrier to adherence. The data collection instrument was first prepared in English and translated into Amharic and then back into English.

### Validity and reliability of the instrument

The reliability of ASK-12 tool was assessed by Cronbach’s alpha coefficient with a value greater than 0.70 was considered as acceptable [19]. The validity of ASK-12 tool was examined by observing the correlation coefficient of the tool with Mini-asthma quality of life questionnaire (Mini-AQLQ). Pearson’s correlation coefficients were used to quantify the validity of the tool with the value of (r = 0.00–0.10); (r = 0.10–0.39); (r = 0.40–0.69); (r = 0.70–0.89); and (r = 0.90–1.00) were interpreted as weak, moderate, strong, and very strong correlations, respectively [20]. Additionally, the validity of the instrument was strengthening by conducting the pre-test among 30 respondents. The results of the validity and reliability of the instrument are shown in the following table (Tables 1 and 2).

**Table 1:** Internal consistency of ASK-12

ASK-12 sub-scale and total scale	Cronbach’s alpha value
Inconvenience/forgetfulness	0.81
Treatment belief	0.77
Behavior	0.76
**ASK-12 total score**	**0.84**

**Table 2:** Spearman’s Correlation of ASK-12 with Mini-AQLQ

ASK-12 subscale and total scale				
	Inconvenience/forgetfulness sub-scale	Treatment belief sub-scale	Behavior sub-scale	ASK-12 total score
Mini-AQLQ total scale	-0.28	-0.24	-0.17	-0.34

*Note: ASK-12* Adherence Starts with Knoladge-12, *Mini-AQLQ* Mini-Asthma Quality of Life Questionnaire

### The data collection procedure

The data were collected by three trained nurse professionals who were working from each three governmental hospitals. Each study participant was identified based on his medical record number. For the study participants who can well read, write, and understand, the questionnaire was given to them to fill out by themselves, and for those illiterate and unable to understand, the data collectors interviewed the study participants based on their date of appointment taken from the registration book. Data were collected after ethical clearance letter was obtained from Addis Ababa University, School of Pharmacy, Ethics Review Committee.

### Data analysis

Descriptive statistics were performed to describe sociodemographic and clinical variables. Frequencies and percentages were used to express categorical variables. The normality of continuous variables was checked by the Shapiro–Wilk test and presented as mean ± standard deviation for normally distributed data and median with interquartile range (IQR) for not normally distributed data. Both bivariable and multivariable logistic regression analyses were performed to identify predictors of non-adherence. In bivariate logistic regression analysis, independent variables with a *P* value less than or equal to 0.25 were entered into multivariate logistic regression analysis to control potential confounding variables that affect medication non-adherence. The variance inflation factor (VIF) was used to detect any evidence of multicollinearity problems among selected independent variables. All statistical tests were analyzed using STATA version 16. The results were considered statistically significant if they had *P* values less than 0.05.

## Results

A total of 422 chronic asthma were systematically selected from the follow-up chest clinics of the three public hospitals in Bahir-Dar city. There was no missing data on the questions given to study participants. The reliability of the 12 elements of ASK-12 tool was evaluated. The results of the reliability test based on the Cronbach alpha value were 0.84, which is higher than the acceptable value. The results of the Pearson correlation coefficient also ranged from 0.17 to 0.34, which meets the criteria of both convergent and divergent validity for the tool.

### Sociodemographic characteristics of the respondents

There were 181 (45.25%) male and 219 (54.75%) female respondents with the median age value of 39 (IQR: 30- 50) years. Most of the respondents were married (323, 76.54%) and lived in urban areas (324, 76.78%). Approximately 385 (91.23%) of the study participants followed orthodox Christianity as their religious affiliation. Regarding educational status, most of the participants had attended higher education. Most of the study participants (32.94%) were government employees and housewives (30.81%). The majority of the study participants’ monthly income was within the range of 1000 to 5000 Ethiopian birr and only 3 (0.71%) of the study participants were active smokers (Table 3).

**Table 3:** Sociodemographic characteristics of TGSH, FHCSH, and Addis Alem Hospital respondents (*N* = 422)

		N	(%)
Gender	Male	193	(45.73)
	Female	229	(54.27)
Age (in years)	Median (first and third quartile) with range	30	50
Marital status	Single	68	(16.11)
	Married	323	(76.54)
	Divorced	17	(4.03)
	Widowed	14	(3.32)
Residence	Rural	98	(23.22)
	Urban	324	(76.78)
Religion	Orthodox	385	(91.23)
	Muslim	21	(4.98)
	Protestant	16	(3.79)
Level education	Unable to read and write	28	(6.64)
	Able to read and write (non-formal educations)	44	(10.43)
	Primary School (1-4^th^)	28	(6.64)
	Primary school (5-8^th^)	58	(13.74)
	Secondary school (9-10^th^)	52	(12.32)
	Secondary school (11-12^th^)	42	(9.95)
	Higher Education (> 12^th^)	170	(40.28)
Employment	Government employee	139	(32.94)
	Employee of private company	46	(10.90)
	Farmer	53	(12.56)
	House wife	130	(30.81)
	Student	12	(2.84)
	Unemployed	7	(1.66)
	Retired	5	(1.18)
	Merchant	30	(7.11)
Monthly income (in Eth Birr)	Less than 1000	149	(35.31)
	In between 1000–5000	200	(47.39)
	Greater than 5000	73	(17.30)
Smoking status	Smoker	3	(0.71)
	Non-smoker	419	(99.29)

### Clinical characteristics of the respondents

Regarding the disease and medication history of the participants, although more than half of the respondents had no family history of asthma, 242 (57.35%) but, about 180 (42.65%) study participants had family history of asthma. Approximately 375 (88.86%) of the study participants had allergic rhinitis and around 127 (30.09%) of the respondents had a comorbid disease. The most frequently encountered comorbid diseases during the data collection period were hypertension (47) (37.01%), diabetes mellitus (33) (25.98%), tuberculosis and pneumonia (9) (7.09%) each. The minimum and maximum disease duration that the patient lived with were 1 and 40 years, respectively, with a mean value of 6.98 ± 6.86. Short-acting beta agonists with inhaled corticosteroids were the most often recommended pharmaceutical combination for treating bronchial asthma during the study period, and there was no evidence of polypharmacy at that time. The clinical characteristics of study participants are shown in (Table 4).

**Table 4:** Clinical characteristics of TGSH, FHCSH and Addis Alem Hospital respondents (*N* = 422)

		N	(%)
Family history of asthma	Yes	180	(42.65)
	No	242	(57.35)
Allergic rhinitis	Yes	375	(88.86)
	No	47	(11.14)
Comorbid disease	Present	127	(30.09)
	Absent	295	(69.91)
Type of comorbid disease	Hypertension	47	(37.01)
	Diabetes	33	(25.98)
	Tuberculosis	9	(7.09)
	Pneumonia	9	(7.09)
	Chronic obstructive disease	7	(5.51)
	Congestive heart failure	6	(4.72)
	Retrovirus infection	6	(4.72)
	Hypertension + diabetes	3	(2.36)
	Others (like chronic kidney disease, hypertension and rheumatoid arteritis, nephrotoxicity, peptic ulcer disease, tuberculosis and pneumonia, stroke)	7	(5.51)
Current asthma medication	Short-acting B2 -agonist alone	55	(13.03)
	Short-acting B2-agonist + inhaled steroid	296	(70.14)
	Short- and long-acting B2-agonist + ICS	71	(16.82)
Disease duration (in years)	Mean ($\pm$ SD)	Minimum	Maximum
	6.98 ± 6.86	1	40

*Note*: *SD* Standard Deviation, *ICS* inhaled corticosteroids

### Adherence status of asthmatic patients

Previous studies determined that 23 was the ideal cutoff value for the ASK-12 total mean score of ASK-12 in asthma patients to identify nonadherence. According to this cut-off value, an asthmatic patient is considered non-adherent to their anti-asthmatic medication if the overall mean score was less than 23 and adherent if the total mean score was above 23 [21]. Around 234 (55.45%) of the study participants in our study were non-adherent to their prescribed medicines. The remaining, 188 (44.55%) respondents, adherent to their anti-asthmatic medicines (Table 5).

**Table 5:** Medication non-adherence status of TGSH, FHCSH and Addis Alem Hospital respondents (*N* = 422)

ASK-12-Am subscale and total score	Mean ± SD			
Inconvenience/forgetfulness	8.54 ± 2.19			
Treatment belief	$8.02\pm$ 1.89			
Behavior	7.21 ± 2.62			
Total	**23.77 ± 4.42**			
Medication non-adherence status using ASK-12	Adherent		Non-adherent	
	N	(%)	n	(%)
	**188**	**(44.55%)**	**234**	**(55.45%)**

*Note*: *ASK-12-Am* Adherence Starting with Knowledge-Twelve-Amharic Version, *SD* Standard Division

### Predictors of nonadherence to anti-asthmatic drugs

As shown in Table 6, analysis of the research data with bivariate logistic regression showed that family history of asthma, duration of the disease, income, educational status, and religious affiliation were significantly associated with patient nonadherence to anti-asthmatic medication. Multicollinerity tests were performed and found that there was no significant correlation between variables. Then, four variables were taken forward to multivariable logistic regression analysis. Finally, three predictors were remained after a backward stepwise selection process. The educational status of the study participants (AOR = 0.03, 95% CI = 0.00–0.05), the family history of asthma (AOR = 0.13, 95% CI = 0.04–0.21) and disease duration that the patients lived (AOR = 0.01, 95% CI = 0.00–0.01) were predictors of non-adherence to antiasthmatic medications for asthmatic patients (Table 6).

**Table 6:** Logistic regression analysis of medication non-adherence and its predictors among asthmatic patients at TGSH, FHCSH and Addis-Alem Hospital (*N* = 422)

Variables	COR, 95% CI	*P*-value	AOR, 95% CI	*P*-value
Educational status	0.85 (0.72–1.00)	0.05	0.03 (0.00–0.05)	0.03
Income	0.75 (0.49–1.15)	0.19	0.04 (0.03–0.11)	0.28
Family history of asthma	0.48 (0.29–0.78)	0.00	0.13 (0.04–0.21)	0.00
Disease duration	1.04 (1.01–1.08)	0.00	0.01 (0.00–0.01)	0.00

*Note*: *AOR* adjusted odd ratio, *COR* crudes odd ratio, *CI* confidence interval

## Discussion

Medication non-adherence has been identified as a significant public health issue as it greatly worsens clinical outcomes and extravagant health-care costs from both the government and public perspectives. Non-adherence to medication in chronic disease is relatively high compared to non-chronic disease and, as patients who continue to take the drug are disappointingly low and drop their medication extremely after the first six months of treatment [22].

Estimating the extent of nonadherence to medicine and investigating the various predictors related to medication non-adherence using various methods are among the areas of concern that require attention, as is strengthening medication adherence. Understanding predictors that significantly affect medication non-adherence is very important to develop effective approaches that improve medication adherence among patients with chronic diseases, including asthma [23]. In this regard, this study explores how to determine the magnitude of nonadherence to medications and its associated factors among asthmatic patients who have active follow-up in the chest clinics of TGSH, FHCSH, and Addis-Alem Hospital.

In our study, the general prevalence of non-adherence to antiasthmatic medication was found to be 55.45%. This level of medication non-adherence implies limitations in the service provided and counselling about the value of strict adherence to their medications. The finding of this study was similar to a study conducted at Soddo Christian General Hospital, Southern Ethiopia (59%), Northern Ethiopia (57%) of asthmatic patients did not adhere to their medication [24, 25].

Identifying the predictors for medication non-adherence is crucial in order to determine the best way to intervene and increase the control of asthma. The results of previous studies indicate that poor adherence has been associated with factors related to the patient's age, lack of knowledge of the disease, medication regimen complexity, drug adverse effects of the drug, and educational level [26, 27].

In this study, the educational status of the respondents, the duration of the disease, and the family history of asthma were the predictors of nonadherence to anti-asthmatic medicines in asthmatic patients. Respondents with a low level of education were associated with poor medication adherence. This finding is supported by previous studies that found that asthmatic patients with an improved level of education showed good adherence [27]. This is due to the fact that patients with a high level of education have a better attitude about treatment and the negative consequences of non- adherence.

Disease duration that the respondents were living with was also another predictor for medication non-adherence in asthmatic patients. Patients with long durations of the disease are more likely to show poor adherence to their medication. This is due to the fact that, as long as the respondents are living with the disease, there is a higher probability of the occurrences comorbid disease that makes the drug regimen more complex and less affordable. Consequently, the level of adherence that the patient shows to their medication is more challenging. This finding was supported by another study conducted in the northern part of Ethiopia [25]. In addition to these, non-adherence to medication in patients with asthma was significantly associated with a family history of asthma. In our study, many of the respondents with uncontrolled asthma who did not adhere well to their medication had a family history of asthma. A similar finding was also reported in a previous study conducted in Uganda [28].

The current study had certain limitations. Asthma can began at any age, but in this study child less than 18 years of age are not included and non-adherence level is not studied. This is the first limitation of the study. The second limitation of the study was the possibility of recall bias since the study uses patient self-reported data. The current study did not show which type of patients are non-adherent to which type of specific drug. This is the third limitation of the study.

## Conclusions

More than half of the study participants in our study demonstrated that they were not adhering to their prescribed anti-asthmatic medications. Educational status of study participants, family history of asthma, and duration of the disease were the predictors of non-adherence of asthmatic patients to their antiasthmatic medications. Therefore, ministry of health, health policy makers, clinicians and other healthcare providers should pay attention to strengthen the adherence level to anti-asthmatic medications and country based interventions should be developed to reduce the burden of non-adherence to anti-asthmatic medications.

## Data Availability

All data generated or analyzed during this study are included in this published article.

## Abbreviations

ASK-12: Adherence Starts with Knowledge-twelve
FHCSH: Felege-Hiwot Comprehensive Specialized Hospital
IQR: Interquartile Range
Mini-AQLQ: Mini-Asthma Quality of Life Questionnaire
TGSH: Tibebe Ghion Specialised Hospital
VIF: Variance Inflation Factor
WHO: World Health Organization
